# Adverse Prognostic Factors and Optimal Intervention Time for Kyphoplasty/Vertebroplasty in Osteoporotic Fractures

**DOI:** 10.1155/2014/925683

**Published:** 2014-01-19

**Authors:** Ioannis D. Papanastassiou, Andreas Filis, Kamran Aghayev, Zinon T. Kokkalis, Maria A. Gerochristou, Frank D. Vrionis

**Affiliations:** ^1^H. Lee Moffitt Cancer Center & Research Institute, Neurooncology Program and Department of Neurosurgery and Orthopaedics, University of South Florida College of Medicine, 12902 Magnolia Drive, Tampa, FL 33647, USA; ^2^General Oncological Hospital “Agioi Anargyroi”, 14564 Athens, Greece; ^3^“Attikon” Hospital, Department of Orthopedics, Athens University Medical School, 12462 Athens, Greece; ^4^“Andreas Syngros” Hospital, 16121 Athens, Greece

## Abstract

*Introduction*. While evidence supports the efficacy of vertebral augmentation (kyphoplasty and vertebroplasty) for the treatment of osteoporotic fractures, randomized trials disputed the value of vertebroplasty. The aim of this analysis is to determine the subset of patients that may not benefit from surgical intervention and find the optimal intervention time. *Methods*. 27 prospective multiple-arm studies with cohorts of more than 20 patients were included in this meta-analysis. We hereby report the results from the metaregression and subset analysis of those trials reporting on treatment of osteoporotic fractures with kyphoplasty and/or vertebroplasty. *Results*. Early intervention (first 7 weeks after fracture) yielded more pain relief. However, spontaneous recovery was encountered in hyperacute fractures (less than 2 weeks old). Patients suffering from thoracic fractures or severely deformed vertebrae tended to report inferior results. We also attempted to formulate a treatment algorithm. *Conclusion*. Intervention in the hyperacute period should not be pursued, while augmentation after 7 weeks yields less consistent results. In cases of thoracic fractures and significant vertebral collapse, surgeons or interventional radiologists may resort earlier to operation and be less conservative, although those parameters need to be addressed in future randomized trials.

## 1. Introduction

Vertebral compression fractures (VCFs) constitute a major health problem with great impact on patient morbidity and mortality [1–3] and almost 1.5 million people are affected annually worldwide [4]. Nonsurgical treatment (NSM) may lead to persisting pain and disability in a significant amount of affected individuals [5] compromising their quality of life [6, 7]. Minimally invasive vertebral augmentation procedures (VAPs) such as balloon kyphoplasty (BKP) and vertebroplasty (VP) have been widely advocated to deal with VCFs. While a 20-year experience and published studies support their utility, three randomized trials (RCTs) comparing VP with a sham procedure [8, 9] or NSM [10] have created contention about the efficacy of VP. Although potential flaws confounding the outcomes have been previously outlined [11–13], they indicate that a subset of patients may not be surgical candidates or have adverse outcome. Location, fracture type, degree of vertebral body collapse and kyphotic angle are identified as possible variables confounding the therapeutic result [14–18].

Another controversial issue is the optimal intervention time; studies with earlier intervention in general yielded superior results [11, 19–22], although good results have been reported up to 3 years after a VCF [23]. Since many patients recover spontaneously, many recommended a few weeks of conservative treatment, provided that adequate pain control has been achieved [13].

To analyze those issues (optimal intervention time and risk factors for suboptimum outcome such as fracture location and collapse) we conducted a metaregression of prospective comparative studies (level I and II data) and also performed a comparison analysis of those studies with the 3 RCTs that reported inferior results. Based on the analyses we attempted to formulate a therapeutic algorithm for the treatment of VCFs.

## 2. Materials and Methods

We recently published our results from a meta-analysis on prospective comparative studies of VAPs for osteoporotic thoracolumbar fractures with cohorts of more than 20 patients [24]. Exclusion criteria were single-arm studies, BKP studies with noninflatable balloons, non-English literature, systematic reviews, meta-analyses, or studies not reporting clinical outcomes and nonosteoporotic fractures. 27 studies were selected [8–10, 20–23, 25–43], which included 8 randomized trials. Some of the studies reported effects from the same patient cohort and were combined (Kasperk/Grafe et al. [20, 21, 23, 25], and Rousing et al. [10, 38]). Mean, SD, and N were required for analysis of continuous variables but were frequently not available. Whenever possible, these were imputed from other summary statistics [44]. For effects measured repeatedly over time (i.e. pain scores), mean differences from baseline were used in a metaregression of days from baseline to assess for time-dependent effects. When the metaregression yielded a nonsignificant slope, we combined multiple time point measures to yield a more precise per-study effect size. If the original scale of measure for an effect could not be preserved, we calculated standardized mean differences [45].

We also tried to elucidate if there is a difference in baseline characteristics in the studies that were related to the different results [8, 9, 38, 39]. In particular, we examined fracture location and fracture severity and related these to the outcomes since they have been implicated as adverse risk factors in other published series [14–18]. Categorical variables were compared using Fisher's exact test and chi-square test was used to detect differences between studies. For pain, metaregression was computed. In all cases, a *P* < 0.05 was selected to evaluate statistical significance. Direct treatment comparisons and outcomes have already been published as part of the present study [24].

## 3. Results

There was a different fracture location distribution in the Rousing study [10, 38] (NSM arm) in comparison with the remaining contributing studies [9, 22, 36] that provided relevant data (*P* = 0.001, Fisher's exact test). The Rousing study had significantly more lumbar fractures than the other studies. More thoracic fractures were treated in the VP arm of Buchbinder et al. [9] (27 thoracic fractures from 45 total fractures), although this did not reach statistical significance (Fisher's exact test, *P* = 0.216). Also the sham group showed a trend towards having fewer thoracic fractures as compared to the VP group (chi-square test, *P* = 0.09).

In regard to fracture severity (mild versus moderate versus severe), data were provided by the Buchbinder [9], INVEST [8] and VERTOS I [39] studies. In Buchbinder et al. [9] and Kallmes et al. [8] there was no difference in the distribution of fracture severity (Chi-Square test, *P* = 0.24 and *P* = 0.19, resp.). On the other hand, there were significantly more severe fractures than in the other available VP arms in VERTOS I (Chi-square with Yates correction, *P* < 0.001).

**Figure 1:**
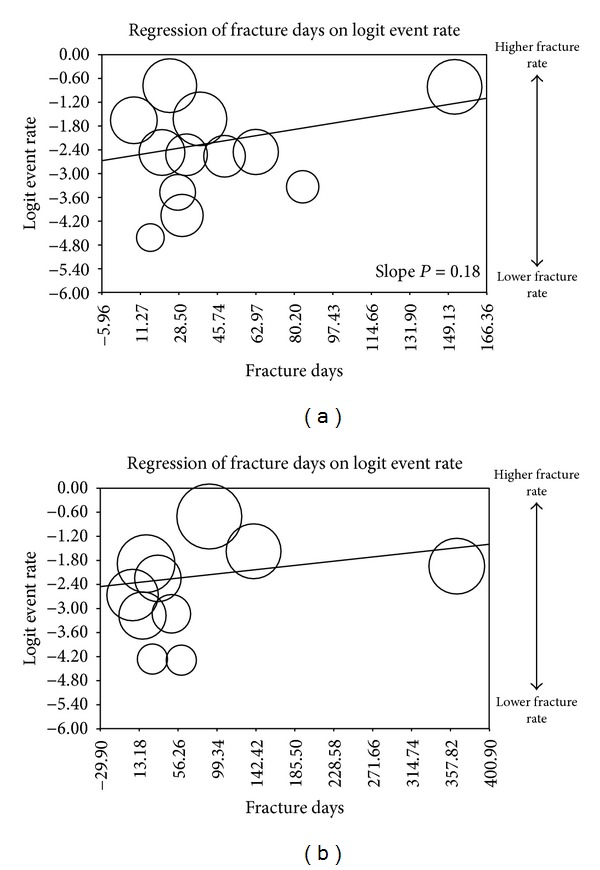
(a) Metaregression of subsequent fracture versus fracture age/VP. (b) Metaregression of subsequent fracture versus fracture age/BKP.

**Figure 2:**
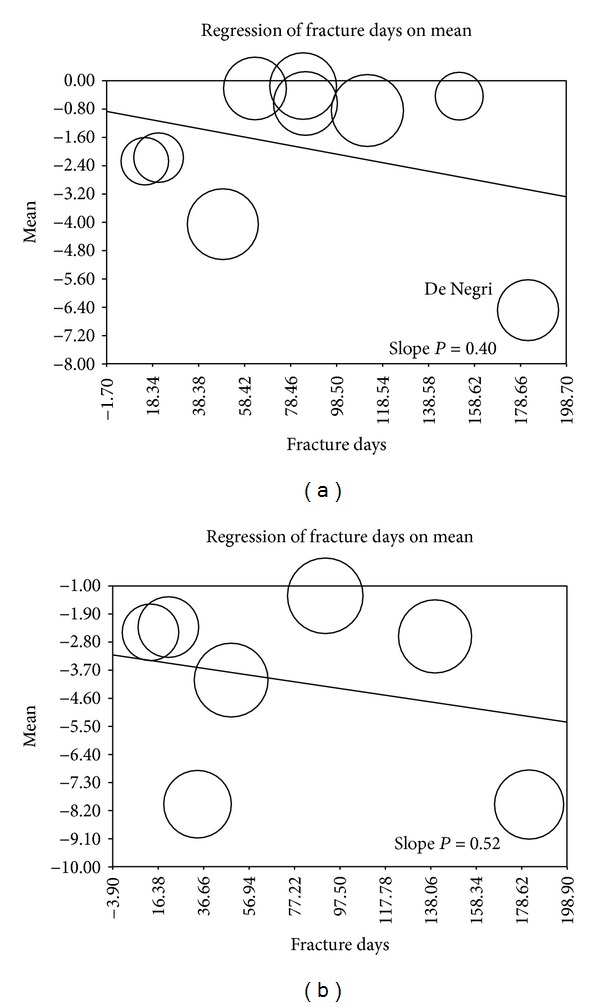
(a) Metaregression of disability versus fracture age/VP. (b) Metaregression of disability versus fracture age/BKP.

**Figure 3:**
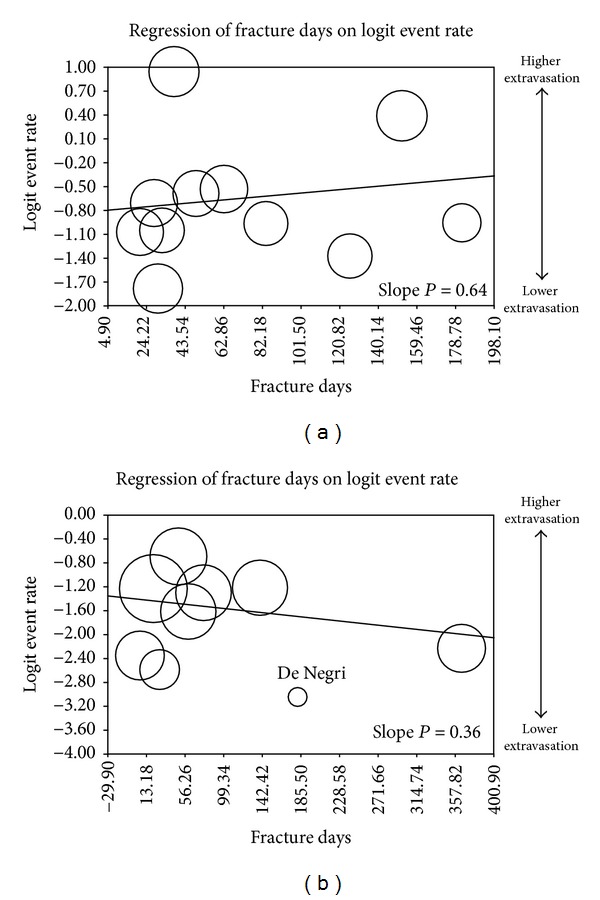
(a) Metaregression of cement extravasation versus fracture age/VP. (b) Metaregression of cement extravasation versus fracture age/BKP.

The sensitivity analyses on average baseline index fracture age against subsequent fractures and disability did not yield significant results (Figures 1 and 2). Cement extravasation for VP had a statistically significant slope (*P* = 0.09) suggesting that older fractures may have a higher extravasation rate. Cement extravasation for BKP did not exhibit a dependence on fracture age (Figures 3(a) and 3(b)).

**Figure 4:**
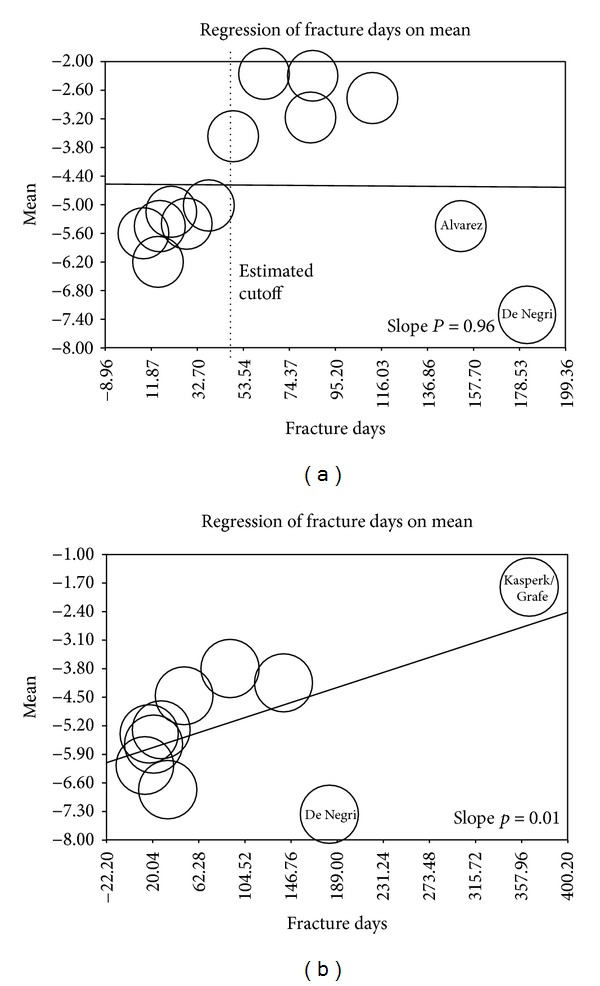
(a) Metaregression of pain reduction versus fracture age/VP group. (b) Metaregression of pain reduction versus fracture age/BKP group.

The metaregression of pain reduction against baseline fracture age exhibited a clear pattern, with clinically significant pain reduction before 7 weeks (~−5.0 to −7.0 points) and substantially less pain reduction between 7 weeks and 4 months, especially for VP (~−2.3 to −3.5 points for VP and ~−3.8 to −4.5 points for BKP) (Figures 4(a) and 4(b)).

## 4. Discussion

Traditionally both VP and BKP have been accepted as successful; however, 2 RCTs that were published in NEJM comparing VP with a sham procedure [8, 9] (along with another randomized trial comparing VP with NSM [10]) have created contention about the efficacy of VP. Inherent problems with those RCTs have been reported: low accrual rate at busy VP centers, which raises the issue that many candidates for VP opted not to participate in the trial; studies not reporting what happened to those patients; sham design; acuity of fractures varied and MR imaging was not used in every case; a large number of screened patients had no fractures and authors in those studies did not use (or did not report using) clinical tests to delineate the pain generator [11–13].

Nevertheless, the 3 RCTs that showed no superiority of VP versus NSM demonstrate that there is a subset of patients selected in these studies that did not benefit as much as anticipated from vertebral augmentation. To identify this patient population we attempted to perform a metaregression analysis on the selected prospective comparative studies and compare them to those trials. The analysis of pain reduction against baseline fracture age exhibited a clear clustering of effects, with clinically significant pain reduction prior to 60 days and much less pain reduction between 60 and 120 days.

**Figure 5:**
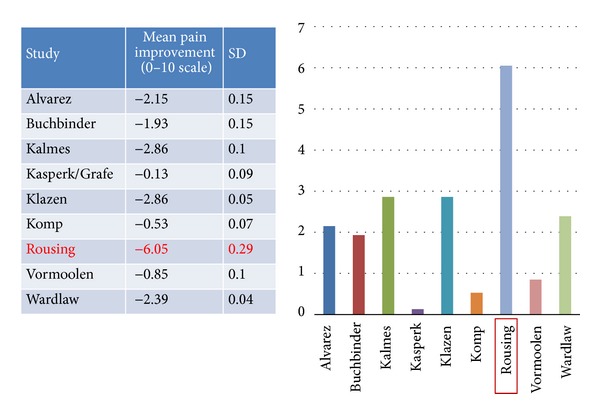
Pain reduction in the NSM group.

In the Danish RCT [10] the striking feature was the significant reduction of pain in the NSM group compared with the NSM arms in the other studies (6.2 versus 2/10 on average) (Figure 5). The subgroup analysis showed that this arm contained significantly fewer thoracic fractures than the rest (*P* < 0.001) creating a potentially more favorable profile. This has been suggested by a previous prospective study, where severe collapse, burst fractures, and thoracic fractures were risk factors for unfavorable outcome after NSM [5] and a study from the SWISS spine registry where lumbar BKPs fared the best [15]. Also the fractures were hyperacute (less than 1 week on average, far less than all other NSM arms). Should the authors delay the surgical intervention in this hyperacute period, where most of the spontaneous improvement is anticipated [5], a significant portion would have escaped the procedure. This phenomenon was also encountered in VERTOS II where more than half of the patients were excluded due to improvement of symptoms during screening [40].

In the other 2 studies we encounter a different scenario. In those studies, the VP arm was not as efficacious as in other studies. Interestingly we could identify 2 clusters of groups in the VP arms: the first consists of 9 studies that report VAS reduction >5 points [10, 28, 29, 31–33, 35, 36, 40] and 3 studies (the 2RCTs [8, 9] plus the VERTOS I [39]) that have only a 50% effect (2.5-point reduction). But why are those 3 studies so distinctly different from the others in the VP group? In terms of the thoracic/lumbar fracture ratio, the Australian sham group exhibited a trend towards having fewer thoracic fractures as compared to the VP group (*P* = 0.09). However, the VP arm had also a potentially less favorable profile when compared with the VP groups in the other studies. Additionally, the severity of VB collapse may have contributed as shown in VERTOS I (more patients with severe collapse than in the other studies).

Therefore, there is indirect evidence that fracture location may contribute to the outcome with better results in the Rousing NSM arm and worse in Buchbinder VP arm. Thoracolumbar fractures are also of special concern, since they pertain to an important biomechanical area with increased stresses and resolution of pain may not be the only goal. Roder et al., in their study from the SWISS spine registry, reported that those fractures had a worse result than the lumbar ones [15]. Unfortunately the studies provide insufficient data to support or refute this notion. Fracture severity may play an additional role, as shown in VERTOS I; as mentioned above both of these parameters were considered dismal prognostic factors in the prospective study by Suzuki et al. [5]. Lee et al. observed prospectively that severe vertebral body collapse (more than 28.5%) led to failure of conservative management [14]. Burst fractures (type A3.1) had about one-third of the probability for average pain relief compared with wedge-impaction injuries (type A1.1) [15]. In this paper Roder et al. observed that cement volume correlated with pain relief, so besides the inherent instability associated with burst fractures, less cement filling volumes may also contribute to inferior postoperative outcomes. Future studies should provide clear baseline demographic data on fracture location and type and severity and analysis of outcomes (pain, disability, and quality of life) in relation to fracture subtypes.

Overall, the worse outcome in the 3 RCTs (along with VERTOS I) seems to be multifactorial. Besides possible issues related to patient selection or trial design as discussed above, the special characteristics of the VCFs treated (location and severity) along with intervention time have possibly influenced the outcomes. Delayed augmentation yields inferior pain relief, whereas, on the other extreme, intervention during the hyperacute period (see Rousing and VERTOS II studies) leads to unnecessary surgery.

## 5. Treatment Algorithm

**Figure 6:**
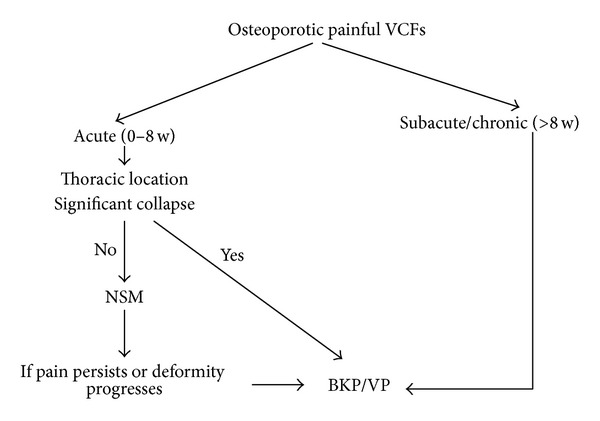
Treatment algorithm.

There is controversy regarding the optimal time of intervention, with some authorities recommending early intervention [46, 47] and others suggesting that late augmentation does not compromise outcome [23, 48]. In our analysis in most of the VP studies that yielded significant pain relief, mean fracture age was less than 7 weeks, while 4 studies with older fractures, including Kallmes/Buchbinder/VERTOS I studies [8, 9, 28, 39], showed suboptimal results. In the BKP group (but not in the VP arm) there was a positive correlation between fracture age and pain relief, but results should be interpreted with caution due to outliers. Based on the observations of our study and also previous suggestions from other authorities [19] we propose a therapeutic “window” period (up to 7 weeks) where the surgeon may adopt a “wait and see” approach without compromising results for favorable fractures, provided that adequate pain control has been achieved. This period differs between authors and societies with recommendations varying from 2 to 6 weeks [14, 25, 33, 49, 50].  Figure 6 shows our proposed therapeutic algorithm. It should be noted, however, that the algorithm is based on the subgroup analysis and data were reported sporadically and not consistently to make a strong correlation.

## 6. Limitations

There are certain limitations to our study, since we have examined predictors at the aggregate or study-level, rather than the experience of individual patients. In the future, identifying person-specific factors that are related to the clinical outcomes will be an important area of inquiry. Moreover, there may have been factors that we were unable to control which may have impacted the pattern of results that were observed. Nevertheless, we were able to identify several features that appear to be important when attempting to resolve the differences across studies.

## 7. Conclusion

Vertebral augmentation in VCFs up to 7 weeks old yields more consistent and superior results, whereas on the other hand surgical intervention in the hyperacute period (up to 2 weeks) should not be pursued. However, it should be stressed that this pertains to the evaluable studies; thoracic location and significant vertebral collapse may be adverse prognostic factors, amongst other confounding variables, and in those cases the operator may choose to proceed earlier in BKP/VP. Careful patient selection is of paramount importance to exclude other pain generators.
